# 

**Published:** 1853-04

**Authors:** 


### CONTENTS.

### ORIGINAL COMMUNICATIONS.

Page.

Address of Dr. Taft before the Mississppi Valley Association of Dental Surgeons, February, 17, 1853.......... 119
A method of forming Sockets for gum teeth on plates. By Dr. J. Taft, Xenia, O,.... 127
On Physicians Extracting teeth. By C. P. Culver, New Castle, Ky., Jan. 3, 1853,... 129
Answer to Dr. Drake's Interrogatories Continued,.......... 133
Minutes of the Second Annual Meeting of the Ohio Dental College Association, held in February, 1853,.......... 142
Ninth Annual Meeting of the Mississippi Vally Association—Held Feb., 17 and 18, 1853, 145
A case of third Dentition. By J. W. Keyes, M. D., D. D. S., of Florida.......... 152
A case of Reproduction of a portion of the Lower Jaw, with the Teeth. By Dr. J. M. Todd, of Monongahela city, Pa.......... 153
Muse on Artificial Teeth,.......... 154
Extraction of Teeth. By Dr. J. Taylor. [Continued.].......... 155

### SELECTED ARTICLES.

Risodontrypy; or Treatment of Exposed Nerves. By C. O. Cone, M. D., of Baltimore,.......... 160

A few weeks Abroad—Notes by the Way,.......... 167

### EDITORIAL.

Risodontropy,.......... 174
The commmencement exercises of the Ohio College of Dental Surgery,.......... 185
Ohio College of Dental Surgery,.......... 176
Philadelphia College of Dental Surgery and New York College of Dental Surgery,.... 176
Prize Essay,.......... 176
The Southern Journal of the Medical and Physical Sciences,.......... 177
Recipe,.......... 187

